# When Simple Orthopedic Cases Become Complex: Case Presentations From Gaza

**DOI:** 10.7759/cureus.75601

**Published:** 2024-12-12

**Authors:** Ahmad Almigdad, Ahmad Bani Salameh, Hamza Abu Hilaleh

**Affiliations:** 1 Department of Orthopedics, Jordanain Royal Medical Services, Amman, JOR; 2 Department of Anesthesiology, Jordanain Royal Medical Services, Amman, JOR

**Keywords:** blast injuries, bone, conflict, gaza, trauma

## Abstract

Orthopedic injuries in Gaza, many of which would be straightforward to manage under normal circumstances, have become increasingly complex and challenging due to ongoing conflict, severe healthcare limitations, and delayed treatment. This review highlights cases of injuries that, if treated promptly, could have been managed with standard protocols but have evolved into complicated and difficult-to-treat conditions. Delayed care, lack of resources, and restricted rehabilitation significantly increase the complexity of treatment and lead to higher rates of complications, and impaired outcomes.

The cases presented illustrate the substantial barriers to effective care, including an overwhelmed healthcare system burdened by a constant influx of new injuries, leaving older cases to receive only basic interventions such as external fixators. Additional challenges include limited access to advanced surgical interventions and implants, prolonged reliance on external fixation, and inadequate physiotherapy services. These conditions emphasize the urgent need for international support to improve Gaza’s medical infrastructure and address the escalating humanitarian crisis effectively.

## Introduction

For more than a year, Gaza has faced sustained conflict, leading to widespread devastation, mass casualties, and a worsening humanitarian crisis [1]. The ongoing violence has significantly strained the healthcare system, with limited medical supplies and damage to healthcare facilities impeding life-saving treatment. These challenges have resulted in numerous untreated injuries, long-term disabilities, and preventable fatalities.

This review underscores how routine injuries, typically manageable under normal circumstances, became complex medical challenges due to delayed treatment in a conflict setting. Factors such as fibrosis, neurovascular damage, contractures, and insufficient rehabilitation compounded the difficulties. The unique focus on straightforward injuries turned severe highlights the profound impact of resource constraints and conflict on healthcare delivery.

## Case presentation

Case 1: Delayed management of a Monteggia fracture

A 58-year-old male sustained a bullet injury to his right forearm eight months prior, resulting in an open Monteggia fracture (ulna fracture with radial head dislocation). Initial treatment included the application of an external fixator and wound dressings. An early postoperative X-ray, as viewed on the patient’s cellphone, demonstrated good fracture alignment. However, over time, the fracture collapsed, and the radial head became dislocated. After the removal of the external fixator, the patient was left with a severe deformity and significant functional limitations of the right upper limb.

**Figure 1:**
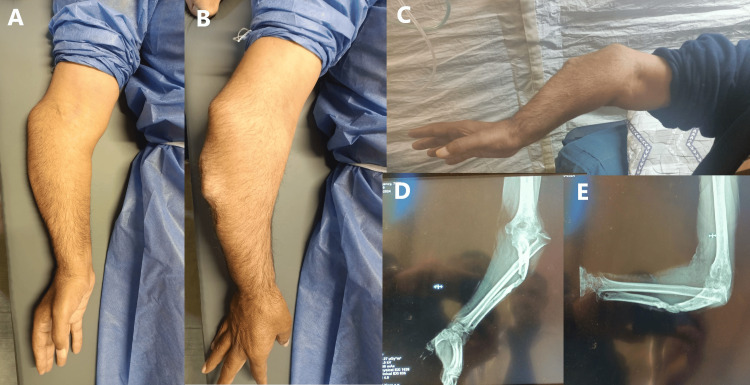
(A, B, C) Preoperative clinical images showing significant deformity, including the prominence of the dislocated radial head and the deformed ulna. (D, E) Anteroposterior and lateral forearm X-rays demonstrate a dislocated radial head, ulnar deformity with bowing, and bone loss. The imaging views are suboptimal due to the limited range of forearm movement.

At presentation, the patient exhibited posterolateral bowing of the forearm with two prominent deformities - one at the radial head and another at the ulna fracture site. Elbow flexion and extension were restricted, with severe limitations in pronation and supination. Additionally, the hand and wrist were stiff, with markedly impaired hand function. Despite the deformities, the neurovascular examination was intact. Radiographic evaluation revealed a posterolateral radial head dislocation, ulna deformity, and evidence of bone loss (Figure 1).

Surgical Intervention

The planned treatment involved radial head excision and corrective osteotomy of the ulna, with the possibility of bone grafting from the fibula. Surgery was performed under general anesthesia with a regional block and a tourniquet. The ulna was exposed via an incision following the deformity. Extensive adhesions were released, and the ulnar nerve was identified in the distal two-thirds of the wound. The nerve was carefully freed from adhesions. The extensor carpi ulnaris tendon was found to be injured, with a significant gap.

At the fracture site, the ulna was refreshed; however, reduction failed due to deformity caused by the dislocated radial head. A direct incision over the prominent radial head allowed its exposure, revealing significant arthritic changes. Initial radial head resection at the neck was insufficient to allow reduction. Consequently, a more proximal resection of approximately 2 cm was performed.

The ulna was then stabilized with a 12-hole dynamic compression plate, and bone grafting was performed using excised portions of the radial head and proximal radius. Intraoperative correction was achieved, and the elbow's range of motion improved significantly. The extensor carpi ulnaris was sutured to its musculotendinous junction, and the wound was closed.

Postoperative Care

**Figure 2:**
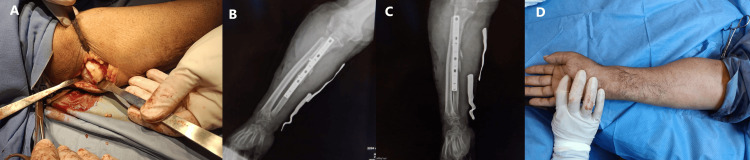
(A) Intraoperative image of radial head exposure showing severe arthritic changes. The radial head and 2 cm of the proximal radius were excised through a direct approach to the dislocated head. (B, C) Anteroposterior and lateral forearm X-rays display ulnar fixation with a fibular graft used to fill the defect, along with radial head excision to facilitate elbow reduction. (D) Postoperative clinical image demonstrating correction of the forearm deformity.

The patient initiated rehabilitation on the second postoperative day to optimize recovery and functional outcomes. However, assessing the final outcomes of healing is often challenging due to patients frequently missing follow-up appointments. This is largely attributed to frequent displacement and the transient nature of many physicians working in Gaza, who typically stay for only a few weeks or months, leaving insufficient time to evaluate long-term results (Figure 2).

Case 2: Blast-induced segmental tibial fracture with bone loss

**Figure 3:**
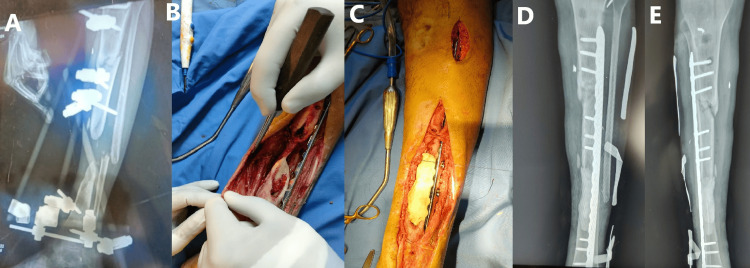
(A) Preoperative leg X-ray showing a segmental tibial fracture with bone loss. An ankle-spanning external fixator is visible, with three pins on the proximal tibia, one on the distal tibia, and a transcalcaneal pin. (B) Intraoperative image displaying significant bone loss, with the posterior tibial nerve visible in the field. (C) Intraoperative image showing fracture fixation using an LCP plate and bone cement to fill the gap as part of the first stage of the Masquelet technique. The plate was inserted percutaneously to span the proximal fracture. A second stage is planned to replace the bone cement with bone graft after six weeks. (D, E) Anteroposterior and lateral leg X-rays showing tibial fracture fixation with bone cement filling the gap. The white shadow surrounding the leg is from radiopaque-marked gauze. LCP: locking compression plate

A 35-year-old male sustained a blast injury to his left leg 10 months prior to presentation, resulting in an open segmental tibial fracture with significant bone loss. An ankle-spanning external fixator was applied at the time of injury (Figure 3). At presentation, the patient’s ankle was stiff and fixed in a plantar flexion position, with rigidly flexed and stiff toes. The vascular examination was intact, but the neurological assessment was limited due to absent ankle and toe movement and altered sensation throughout the leg. Multiple pin-site infections were noted.

Given the pin-site infections, the external fixator was removed from the clinic, and a full cast was applied. The patient was scheduled for the first stage of the Masquelet technique two weeks later to allow pin-site infections to heal.

Surgical Intervention

The surgery was performed under spinal anesthesia with a regional block. An anterolateral approach to the tibia revealed the interposition of anterior and posterior leg compartments with extensive fibrosis. Soft tissue dissection exposed a significantly contracted posterior compartment with fibrotic muscles, suggesting the possibility of an undiagnosed compartment syndrome or fibrotic healing of severe muscle injury.

Attempts at soft tissue release and manipulation of the ankle and toes failed to restore any movement. The fracture site was refreshed, and the longest available plate, a 17-hole 4.5 mm broad locking compression plate (LCP), was applied to span the proximal fracture. A bone cement spacer was placed to prepare for the second stage of the Masquelet technique. The wound was closed without tension, and the patient was observed for signs of compartment syndrome postoperatively.

Postoperative Care

The patient remained comfortable postoperatively and was discharged two days later. He is scheduled for the second stage of the Masquelet technique, which will include bone grafting and potential soft tissue procedures based on his response to physiotherapy.

Case 3: Blast injury to the forearm with segmental bone and soft tissue damage

**Figure 4:**
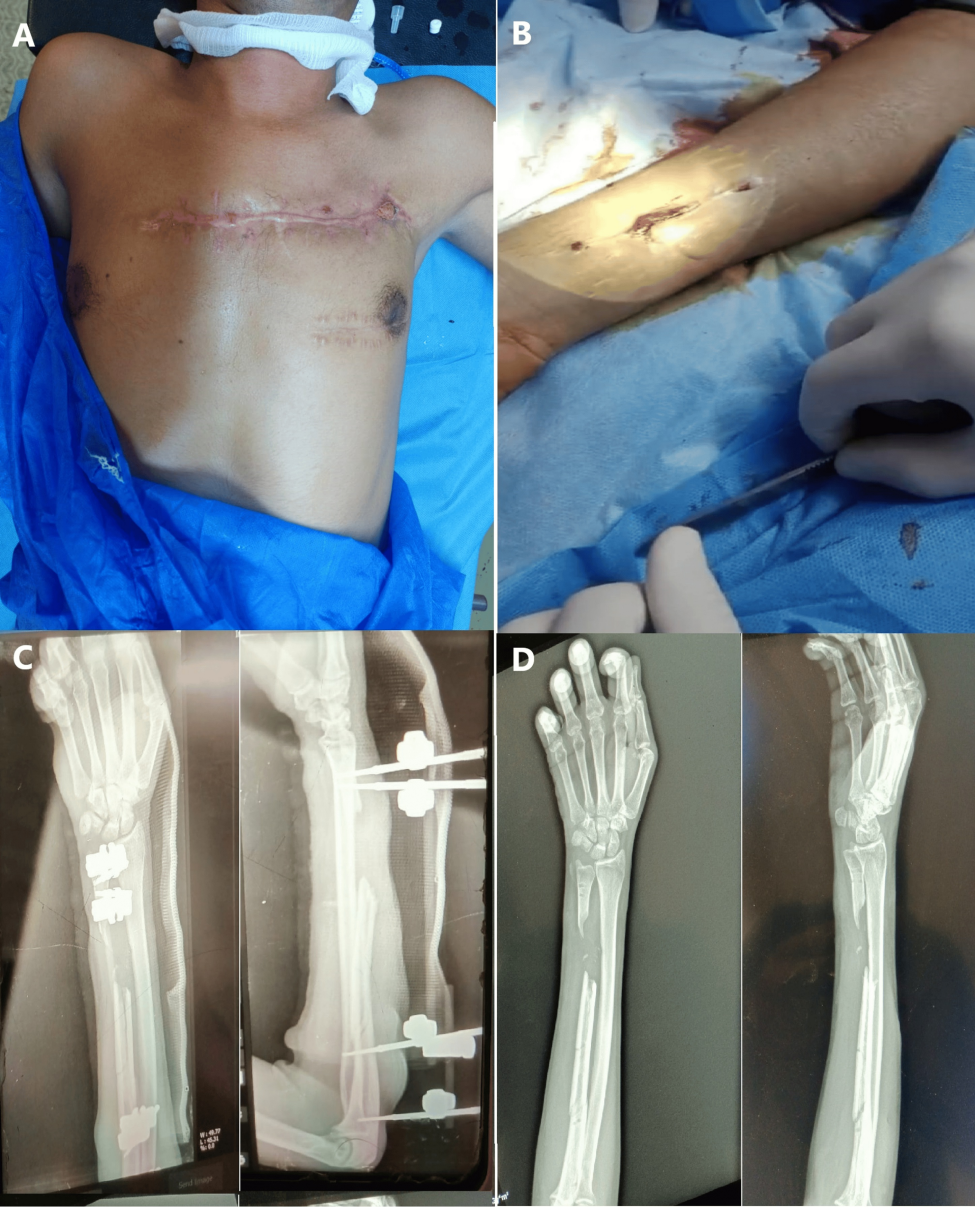
(A) Clinical image of the chest showing two transverse wounds, one at the level of the heart apex, with visible pulsations at the wound site. There is no documentation regarding the extent of the injury, and echocardiogram or chest CT scan was unavailable. A chest X-ray was unremarkable, adding to the complexity of management. As a result, the patient underwent surgery under regional anesthesia. (B) Clinical image of the forearm showing longitudinal wound on the volar aspect. Finger movement caused visible movement of the wound due to tethering of the flexor tendons. (C) Anteroposterior and lateral forearm X-rays showing a segmental ulnar bone fracture with bone loss, stabilized by an external fixator and a splint. (D) X-rays taken after the removal of the external fixator reveal the segmental ulnar bone fracture and associated bone loss.

A 31-year-old male sustained a blast injury to his right forearm two months prior to presentation, resulting in segmental bone loss of the ulna. The radius was intact, but an external fixator had been applied to the ulna. There were dorsal and volar forearm wounds (Figure 4).

At presentation, the patient exhibited rigid finger flexion. Attempts to extend the fingers caused tethering of the flexor tendons at the volar forearm wound. The radial pulse was palpable. Neurological examination revealed decreased sensation across the radial, median, and ulnar nerve distributions, along with weak wrist and finger extension. There were flickering finger movements, but significant tethering at the volar wound was noted. CT angiogram and nerve conduction studies were unavailable for further vascular and neurological evaluation.

The treatment plan involved the removal of the external fixator, fixation of the ulna using a fibular graft or the first stage of the Masquelet technique, and exploration of the volar wound for suspected adhesions around the flexor tendons.

The patient also had a large transverse chest wound with visible cardiac pulsations, reportedly from multiple rib fractures and a prior chest tube application. Documentation regarding potential pericardial or pulmonary injuries was unavailable. Preoperative discussions with anesthesiology were conducted, considering the lack of echocardiography and CT angiogram facilities. A regional block was planned for the upper limb and right lower limb to harvest the fibular graft.

Surgical Intervention

**Figure 5:**
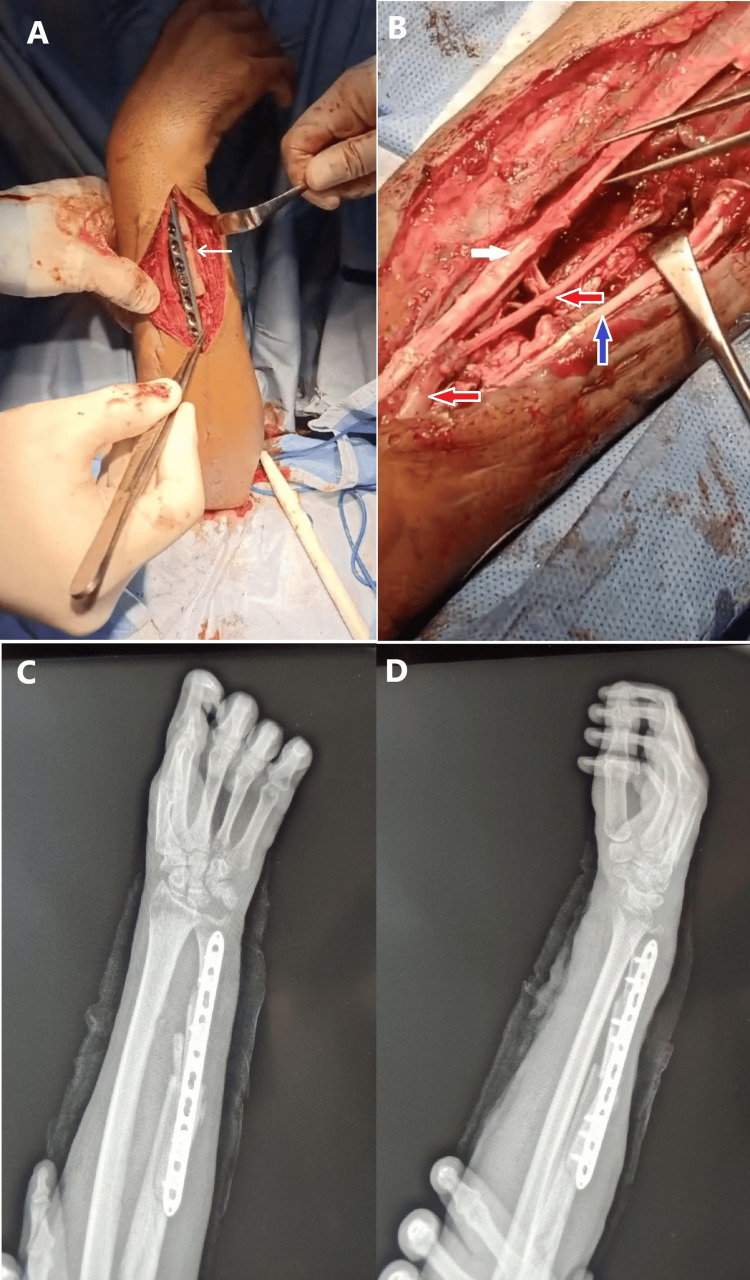
(A) Intraoperative image showing ulnar fixation using a dorsal locking compression plate (LCP) with the gap filled using a fibular graft. The ulnar nerve is visible in the surgical field (arrow). (B) Intraoperative image after exploration of the volar forearm wound, revealing a 7 cm gap in the median nerve and injuries to the flexor tendons (FDS, FDP, FI). The median nerve was reconstructed using cutaneous nerves harvested from the forearm (red arrow), while the flexor tendon gaps were bridged using half of the FCR tendon (blue arrow); the white arrow indicates the FCR. (C, D) Postoperative forearm X-rays show ulnar fixation with the fibular graft in place. The longest available LCP was utilized, though it did not span the proximal fracture, which was confirmed to be healed both clinically and radiologically during surgery. FDS: flexor digitorum superficialis; FDP: flexor digitorum profundus; FI: flexor indicis; FCR: flexor carpi radialis

The ulna was exposed and exploration of the ulnar nerve revealed the absence of nerve injury. The fracture site was refreshed, and a fibular graft was harvested. The graft was secured with a 10-hole 3.5 mm LCP and screws (Figure 5). Volar wound exploration revealed a large fibrous mass with extensive adhesions. Several injuries were identified, including a 7 cm gap in the median nerve and damage to the flexor digitorum profundus (FDP), flexor digitorum superficialis (FDS), flexor indicis, and palmaris longus tendons. The adhesions were released, and the tendons were freed to restore finger extension. To address the tendon injuries, half of the flexor carpi radialis (FCR) tendon was harvested to bridge the gaps in the FDP and FDS tendons, which were repaired as a single mass. The median nerve gap was reconstructed using cutaneous nerves from the forearm. The patient was encouraged to begin immediate physiotherapy to prevent tendon fibrosis and tethering.

Postoperative Care and Future Planning

The patient is currently undergoing a rehabilitation program and may require additional soft tissue procedures depending on his progress. Physiotherapy focuses on maintaining tendon mobility and minimizing further adhesions to optimize hand function.

## Discussion

Orthopedic injuries caused by blasts and bullets are severe and typically require comprehensive, multidisciplinary care involving orthopedic, vascular, and plastic surgery teams, along with psychological and rehabilitative support [2]. Such injuries can occur anywhere in the world. However, the ongoing conflict has severely strained Gaza’s healthcare system, leaving patients with only basic care, such as wound closure and external fixation [3,4]. Advanced interventions, such as nerve or tendon repair and reconstructive surgeries, remain largely inaccessible. As a result, patients often receive incomplete treatment, leading to long-term disabilities and, in many cases, preventable amputations. Moreover, delays in evacuating patients from the scene of injury exacerbate these challenges. Patients often remain trapped under rubble for prolonged periods or face delays in transfer to hospitals due to ongoing conflict and lockdowns. These delays transform simple injuries into complex cases involving contamination, increased risk of vascular injuries, and compartment syndrome.

The lack of timely, specialized care exacerbates complications such as infections, contractures, and fibrosis, leading to chronic disabilities and limited functionality [5]. These challenges, compounded by resource shortages and high patient volumes, leave medical staff struggling to manage even the most basic treatments, often prioritizing stabilization over more complex interventions. As a result, recovery is delayed, future surgeries become more complex, and outcomes are significantly diminished.

The unavailability of appropriate orthopedic implants in Gaza often necessitates the use of suboptimal fixation methods, which significantly compromises patient outcomes. For example, external fixators are sometimes employed as definitive treatment for closed simple fractures, such as neck or femur fractures or long bone fractures, which would typically require internal fixation. Similarly, Kirschner wires are used to stabilize complex, comminuted fractures like humeral head fractures, which is far from ideal. The prolonged use of external fixators, often extending over several months, frequently leads to complications such as pin site infections, malunion, nonunion, and joint stiffness. Many patients suffer from combined injuries, such as the ipsilateral femur and tibia fractures, where extended use of knee-spanning external fixators can result in severe knee stiffness, rendering patients functionally disabled.

Additionally, the lack of timely soft tissue management at the time of injury leads to the development of adhesions and contractures, which become significant challenges for both treating physicians and patients. Many injuries involve bone loss with soft tissues, nerves, and blood vessels interposed in the defect, further complicating surgical management. Rehabilitation remains a critical issue. Even for patients who receive proper surgical treatment, the lack of accessible physiotherapy services severely compromises recovery. Post-injury stiffness is exceedingly common, significantly affecting functionality and quality of life.

Presenting these cases does not highlight rare conditions but rather demonstrates how straightforward injuries can become significantly complicated due to delayed or inadequate treatment. These cases are merely examples; the majority of injuries in Gaza are initially straightforward but become complicated due to improper initial management or delayed treatment. The unavailability of proper implants often leads to the use of unsuitable alternatives, such as external fixators or pins, which increases the risk of nonunion, malunion, infection, and poor outcomes.

In Case 1, a Monteggia fracture would typically be treated with a reduction of the radial head and fixation of the ulna, leading to an expected recovery. However, leaving the radial head dislocated for eight months and failing to reduce the ulna resulted in severe complications, including extensive contractures, fibrosis, and impaired rehabilitation. These factors contributed to joint and finger stiffness, making the surgical intervention more complex, time-consuming, and risky, with a suboptimal outcome. Additionally, the patient required a prolonged recovery period and extensive rehabilitation to address the stiffness and restore functionality.

In Case 2, the segmental tibial bone loss caused by a blast injury underscores the severity of the associated soft tissue damage. Such injuries necessitate timely and staged surgical interventions to address both bone and soft tissue comprehensively. Bone defects can be treated acutely with bone grafting or through Masquelet's induced membrane technique, a two-stage surgical procedure introduced by Dr. Masquelet in the mid-1980s. This technique involves creating an autologous foreign-body membrane in the first stage by implanting a polymethyl methacrylate (PMMA) bone cement spacer, which supports the morselized bone graft implanted during the second stage, promoting graft-to-bone union [6,7].

In this case, the prolonged use of an ankle-spanning external fixator for 10 months, combined with significant bone loss, led to fibrotic tissue formation at the fracture site. The extended duration also resulted in a rigidly plantarflexed ankle and stiffly flexed toes, possibly due to undiagnosed compartment syndrome or extensive fibrosis and contractures in the posterior compartment. Pin-site infections further complicated the situation, increasing the risk of surgical site infections and delaying definitive treatment. Even after achieving bone healing, soft tissue issues remain challenging and may require additional surgeries, such as soft tissue release, tendon transfers, or ankle arthrodesis. This case illustrates the complexity of managing blast-induced injuries, often requiring multiple surgical procedures to address both the initial trauma and its long-term consequences.

In Case 3, the segmental ulna bone loss raised concerns about a possible ulnar nerve injury. However, the presence of rigid fingers and wrist, altered sensation, and tethered flexor tendons with extensive fibrosis made preoperative neurological assessment challenging. In the absence of diagnostic tools such as nerve conduction studies, the surgeon had to rely on surgical exploration. Interestingly, while an ulnar nerve injury was anticipated, the exploration revealed a 7 cm segmental loss in the median nerve instead. Additionally, the flexor tendons were found to be injured, with significant gaps, although the patient retained limited finger flexion due to fibrosis bridging the proximal and distal portions of the flexor muscles. This case illustrates how blast or bullet injuries can cause unexpected damage depending on the trajectory of the projectile through the limb. The associated chest wound further complicated perioperative management, as preoperative imaging tools like chest CT or echocardiography were unavailable, increasing the risks during surgery. Although the interventions addressed immediate issues, the patient may require additional soft tissue procedures, emphasizing the complex, prolonged, and staged nature of recovery in such cases.

Additionally, many procedures are performed by visiting doctors who stay for only a few weeks to a few months. While their contributions are invaluable in addressing immediate needs and providing specialized care, the transient nature of their involvement poses significant challenges in ensuring continuity of care. Patients often require staged surgeries and long-term follow-up, which can be difficult to coordinate once visiting doctors leave. This lack of continuity may result in incomplete treatment plans, delays in addressing complications, or inadequate rehabilitation, ultimately affecting patient outcomes.

The dire circumstances highlight the urgent need for international support to improve Gaza’s medical infrastructure and provide the resources necessary for effective trauma care and rehabilitation. Without this support, the cycle of inadequate treatment, preventable disabilities, and prolonged suffering will persist, underscoring the critical importance of addressing this humanitarian crisis.

## Conclusions

The cases presented in this review highlight the profound impact of resource limitations, conflict-induced delays, and insufficient medical infrastructure on orthopedic care in Gaza. Injuries that are typically manageable with standard protocols have evolved into complex medical challenges, resulting in long-term disabilities and a diminished quality of life for patients. The reliance on external fixation, delayed definitive treatment, and inadequate rehabilitation services underscores the urgent need for enhanced medical resources and infrastructure. Addressing these challenges requires international support to alleviate the growing humanitarian crisis, improve access to specialized care, and optimize outcomes for patients living in conflict zones.
